# Intraoperative detection of ^18^F-FDG-avid tissue sites using the increased probe counting efficiency of the K-alpha probe design and variance-based statistical analysis with the three-sigma criteria

**DOI:** 10.1186/1471-2407-13-98

**Published:** 2013-03-04

**Authors:** Stephen P Povoski, Gregg J Chapman, Douglas A Murrey, Robert Lee, Edward W Martin, Nathan C Hall

**Affiliations:** 1 Division of Surgical Oncology, Department of Surgery, Arthur G. James Cancer Hospital and Richard J. Solove Research Institute and Comprehensive Cancer Center, The Ohio State University Wexner Medical Center, 43210, Columbus, OH, USA; 2 Department of Electrical and Computer Engineering, The Ohio State University, 43210, Columbus, OH, USA; 3 Department of Radiology, The Ohio State University Wexner Medical Center, 43210, Columbus, OH, USA

**Keywords:** F-fluorodeoxyglucose, Image-guided surgery, Radioguided surgery, Gamma detection probes, Positron emission tomography, Neoplasms, Intraoperative detection, Limit of detection, Counting efficiency, T/B ratio

## Abstract

**Background:**

Intraoperative detection of ^18^F-FDG-avid tissue sites during ^18^F-FDG-directed surgery can be very challenging when utilizing gamma detection probes that rely on a fixed target-to-background (T/B) ratio (ratiometric threshold) for determination of probe positivity. The purpose of our study was to evaluate the counting efficiency and the success rate of *in situ* intraoperative detection of ^18^F-FDG-avid tissue sites (using the three-sigma statistical threshold criteria method and the ratiometric threshold criteria method) for three different gamma detection probe systems.

**Methods:**

Of 58 patients undergoing ^18^F-FDG-directed surgery for known or suspected malignancy using gamma detection probes, we identified nine ^18^F-FDG-avid tissue sites (from amongst seven patients) that were seen on same-day preoperative diagnostic PET/CT imaging, and for which each ^18^F-FDG-avid tissue site underwent attempted *in situ* intraoperative detection concurrently using three gamma detection probe systems (K-alpha probe, and two commercially-available PET-probe systems), and then were subsequently surgical excised.

**Results:**

The mean relative probe counting efficiency ratio was 6.9 (± 4.4, range 2.2–15.4) for the K-alpha probe, as compared to 1.5 (± 0.3, range 1.0–2.1) and 1.0 (± 0, range 1.0–1.0), respectively, for two commercially-available PET-probe systems (P < 0.001). Successful *in situ* intraoperative detection of ^18^F-FDG-avid tissue sites was more frequently accomplished with each of the three gamma detection probes tested by using the three-sigma statistical threshold criteria method than by using the ratiometric threshold criteria method, specifically with the three-sigma statistical threshold criteria method being significantly better than the ratiometric threshold criteria method for determining probe positivity for the K-alpha probe (P = 0.05).

**Conclusions:**

Our results suggest that the improved probe counting efficiency of the K-alpha probe design used in conjunction with the three-sigma statistical threshold criteria method can allow for improved detection of ^18^F-FDG-avid tissue sites when a low *in situ* T/B ratio is encountered.

## Background

Intraoperative gamma probe detection of various radioisotopes during radioguided surgery has become commonplace and is an established discipline within the practice of surgery [1]. Along these lines, ^18^F-fluorodeoxyglucose (^18^F-FDG), which is widely used for diagnostic positron emission tomography (PET) imaging for a variety of solid malignancies, has recently become the object of increased investigations into its utility for the identification of ^18^F-FDG-avid tissue sites during radioguided surgery [2-12]. In this specific regard, it has become increasingly advantageous to specifically design intraoperative radiation detection probes to directly or indirectly detect the resultant 511 KeV gamma emissions following positron annihilation emanating from ^18^F-FDG-avid tissues. Nevertheless, most gamma detection probes that are currently commercially available have been designed for detecting radioisotopes of gamma-ray energies much lower than 511 KeV. Such radioisotopes include: (1) ^99m^Tc (140 and 142 KeV) that has most commonly been used for sentinel lymph node biopsy procedures and parathyroid surgery; (2) ^111^In (171 and 247 KeV) that has been used with octreotide to detect neuroendocrine tumors; (3) ^123^I (159 KeV) that has been used with metaiodobenzylguanidine to detect neuroblastomas and pheochromocytomas; and (4) ^125^I (35 KeV) that has been used with anti-TAG-72 monoclonal antibodies and anti-CEA monoclonal antibodies during radioimmunoguided surgery [1].

The success of detecting and localizing ^18^F-FDG-avid tissue sites during ^18^F-FDG-directed surgery is affected by several factors, including: (1) the counting efficiency of the detection probe used; and (2) the target-to-background (T/B) ratio of the radioactive emissions of ^18^F-FDG. Various authors have examined the role played by the T/B ratio for correctly identifying ^18^F-FDG-avid tissue sites for PET imaging [13] and during ^18^F-FDG-directed surgery [14-20]. The finding of a low T/B ratio of ^18^F-FDG is multifactorial, and can be influenced by factors such as the paucity of tumor vascularization, the co-existence of large areas of tumor necrosis, the existence of an intrinsic low metabolic rate for some tumors, and the close proximity of tumor to areas of elevated physiologic ^18^F-FDG uptake or accumulation [1,16-20]. Gulec et al. [16-18] has suggested that a minimum *in situ* T/B ratio of 1.5-to-1.0 for ^18^F-FDG is necessary, in order “for the operating surgeon to be comfortable that the difference between tumor and normal tissue are significant” during ^18^F-FDG-directed surgery. However, it has been our own experience that the observed *in situ* T/B ratio seen during ^18^F-FDG-directed surgery is commonly less than 1.5-to-1.0, and is highly dependent upon the specific detection probe used. Therefore, the *in situ* intraoperative detection and localization of ^18^F-FDG-avid tissue sites during ^18^F-FDG-directed surgery can be very challenging when utilizing standard gamma detection probes and PET probes that rely solely on a fixed T/B ratio (i.e., ratiometric threshold) as the threshold for probe positivity for the identification of ^18^F-FDG-avid tissue sites.

In this regard, it is our contention that improved *in situ* intraoperative detection of ^18^F-FDG-avid tissue sites with a gamma detection probe system can be attained by taking advantage of the increased probe counting efficiency offered by the K-alpha probe design [21] and by utilizing a variance-based statistical analysis schema [22] with the three-sigma criteria [23,24].

A variance-based statistical analysis schema was previously described by Currie for qualitative detection and quantitative determination in radiochemistry [22]. By applying hypothesis testing, Currie reduced the threshold for a significant difference between background radiation and target radiation to a variance-based statistical model. Such hypothesis testing and statistical modeling has become commonplace in the analysis of medical data, including medical imaging [25,26]. The application of variance-based modeling to the determination of the threshold for gamma detection probe positivity, in the form of the three-sigma criteria for gamma detection probe positivity, was popularized by Thurston [23,24] and has since then been well validated in radioimmunoguided surgery involving ^125^I- labeled anti-TAG-72 monoclonal antibodies [24,27-31]. The three-sigma criteria defines a tissue as being probe positive when the count rate in that tissue exceeds three standard deviations above the count rate detected with normal adjacent background tissue [23,24,27-31].

An example of a gamma detection probe that can greatly benefit from the three-sigma statistical threshold criteria is the K-alpha probe [21]. The K-alpha probe design, which was also elucidated by Thurston in 2007, utilizes the concept of detecting secondary, lower energy gamma emissions (K-alpha x-ray fluorescence) that result when a thin metal foil plate (typically lead) is placed between a cadmium-zinc-telluride crystal and a source of gamma emissions, such as ^18^F-FDG [21]. It is our contention that when concurrently utilized, the K-alpha probe design and the three-sigma criteria can improve the intraoperative detection of ^18^F-FDG-avid tissue sites, even at very low T/B ratios for ^18^F-FDG, and would represent a methodology that is superior to a fixed T/B ratio (i.e., ratiometric threshold) methodology used by other gamma detection probe systems for detection of ^18^F-FDG-avid tissue sites.

In the current report, we evaluated the probe counting efficiency and the success rate of *in situ* intraoperative detection of ^18^F-FDG-avid tissue sites (using the three-sigma statistical threshold criteria method and the ratiometric threshold criteria method) that were assessed concurrently with three gamma detection probe systems (consisting of the K-alpha probe system and two commercially-available PET-probe systems) during ^18^F-FDG-directed surgery.

## Methods

All data analyzed in this manuscript were obtained from the master database of an institutional review board (IRB)-approved, prospective, pilot study protocol for multimodal imaging and detection performed during ^18^F-FDG-directed surgery for known or suspected malignancy at the Arthur G. James Cancer Hospital and Richard J. Solove Research Institute of The Ohio State University Wexner Medical Center that was previously approved by the Cancer IRB of the Office of Responsible Research Practices of The Ohio State University.

From a total of 65 patients who gave informed consent to participate in the IRB-approved, prospective, pilot study protocol, a total of 60 patients were taken to the operating room, and of which 58 patients underwent ^18^F-FDG-directed surgery for known or suspected malignancy using gamma detection probes. Of those 58 patients undergoing ^18^F-FDG-directed surgery for known or suspected malignancy using gamma detection probes, we identified all cases in which ^18^F-FDG-avid tissue sites were identified on same-day preoperative diagnostic PET/CT imaging, and for which each of these ^18^F-FDG-avid tissue sites underwent attempted *in situ* intraoperative detection (based upon determination of the *in situ* counts per second measurements recorded during ^18^F-FDG-directed surgery) concurrently using three separate gamma detection probe systems, and then were subsequently surgical excised. The first system was the K-alpha probe system [21]. The two other systems represented commercially-available PET-probe systems that were designed specifically to directly or indirectly detect resultant 511 KeV gamma emissions following positron annihilation emanating from ^18^F-FDG-avid tissue sites. These two commercially-available PET-probe systems were the RMD Navigator™ Gamma-PET™ probe system (RMD PET probe; Dynasil Corporation, Watertown, MA) and the Neoprobe® neo2000® GDS PET probe system (Neoprobe PET probe; Devicor Medical Products, Incorporated, Cincinnati, OH). All three gamma detection probe systems had to be used concurrently in each case for attempted *in situ* intraoperative detection in order for any particular case to qualify for inclusion in the current analyses.

In each instance, a count rate (i.e., counts per second) was taken from an area selected for the measurement of background tissue count rate and from the area of presumed ^18^F-FDG-avid tissue selected for the measurement of target tissue count rate. An area of presumed normal tissue within a region adjacent to the area of the target tissue was selected for the measurement of background tissue count rate. Three separate recorded values were used to generate each averaged target tissue count rate measurement determined for each area of presumed ^18^F-FDG-avid tissue. All values used for the averaged count rate measurements were reported as averaged counts per second. All of the averaged target tissue count rate measurements that are reported in this paper represent measurements taken on an area of presumed ^18^F-FDG-avid tissue before it was surgically excised (i.e., *in situ* measurements). None of the averaged target tissue count rate measurements that are reported in this paper represent measurements taken on an area of presumed ^18^F-FDG-avid tissue after it was surgically excised (i.e., *ex situ* measurements).

The counting efficiency [32] of each of the three gamma detection probe systems was calculated for each ^18^F-FDG-avid tissue site identified during *in situ* intraoperative detection. The probe counting efficiency was defined as a relative probe counting efficiency ratio for each of the individual three gamma detection probe systems, consisting of the ratio of the averaged target tissue count rate for each ^18^F-FDG-avid tissue site using each of the individual three gamma detection probe systems as compared to the averaged target tissue count rate of the gamma detection probe system with the lowest averaged target tissue count rate for each ^18^F-FDG-avid tissue site. Thus, the relative probe counting efficiency ratio for the gamma detection probe system with the lowest averaged target tissue count rate will resultantly be reported as 1.0.

A calculated fixed T/B ratio was calculated for each target tissue as the ratio of the averaged target tissue count rate to the background tissue count rate. A calculated three-sigma criteria count rate was calculated for each target tissue by the methodology popularized of Thurston [23,24], based upon taking the standard deviation derived from the normal background tissue count rate and multiplying that standard deviation by a factor of three and then adding that number to the normal background tissue count rate. For the calculated fixed T/B ratio method (i.e., ratiometric threshold criteria method), a ratiometric threshold of 1.5-to-1.0 or greater was set as the ratiometric threshold criteria of probe positivity. For the calculated three-sigma criteria count rate method, three-sigma statistical threshold of probe positivity was met when the calculated three-sigma criteria count rate for the target tissue was exceeded by the actual target tissue count rate. The determination of probe positivity for successful *in situ* intraoperative detection of ^18^F-FDG-avid tissue sites by each of the three gamma detection probe systems was then compared both by the ratiometric threshold criteria method and by the three-sigma statistical threshold criteria method.

All results were expressed as mean (± SD, range). The software program IBM SPSS® 19 for Windows® (SPSS, Inc., Chicago, Illinois) was used for the data analysis. All mean value comparisons were made by one-way analysis of variance (ANOVA). All categorical variable comparisons were made using 2 × 2 or 2 × 3 contingency tables that were analyzed by either the Pearson chi-square test or the Fisher exact test, when appropriate. Categorical variable comparisons were made for probe type as a function of threshold criteria and for threshold criteria as a function of probe type. P-values determined to be 0.05 or less were considered to be statistically significant. All reported categorical variable comparisons P-values were two-sided.

## Results

Of those 58 patients undergoing ^18^F-FDG-directed surgery for known or suspected malignancy using gamma detection probes, we identified seven patients (four Caucasian males, two Caucasian females, and one African-American female) who underwent same-day preoperative diagnostic PET/CT imaging and in whom all three previously described gamma detection probe systems were then concurrently utilized for attempted *in situ* intraoperative identification of ^18^F-FDG-avid tissue sites between the dates of March 3, 2009 and March 19, 2009. These seven patients had a mean age of 57 (± 12, range 41–80) years, a mean body weight of 79.8 (± 16.8, range 59.9–102.1) kilograms or 176 (± 37, range 132–225) pounds, and a mean same-day pre-scanning blood sugar of 99 (± 21, range 78–137) milligrams per deciliter. The mean ^18^F-FDG injection dose was 540 (± 51, range 433–587) MBq or 14.6 (± 1.4, range 11.7–15.9) millicuries.

Within this group of seven patients, a total of nine separate ^18^F-FDG-avid tissue sites, which were identified on same-day preoperative diagnostic PET/CT imaging, were intraoperatively assessed *in situ* with all three gamma detection probe systems, and were subsequently surgical excised. Additionally, in one of the seven patients, there were four intraoperative clinically suspicious sites (i.e., intraoperative clinically palpable sites) within the surgical field that were not ^18^F-FDG-avid on preoperative same-day diagnostic PET/CT imaging, but were intraoperatively assessed *in situ* with all three gamma detection probe systems and were subsequently surgical excised.

The nine separate ^18^F-FDG-avid tissue sites had a mean SUVmax of 8.6 (± 3.8, range 1.9–13.4) on same-day preoperative diagnostic PET/CT imaging. The mean time from ^18^F-FDG injection to same-day preoperative diagnostic PET/CT imaging in the seven patients evaluated was 94 (± 38, range 66–179) minutes, with only one patient exceeding mean time of 94 minutes from ^18^F-FDG injection to same-day preoperative diagnostic PET/CT imaging. The mean time from ^18^F-FDG injection to the time of the start of surgery in the seven patients evaluated was 219 (± 61, range 168–305) minutes. The mean time from ^18^F-FDG injection to the time of attempted *in situ* intraoperative gamma probe detection in the seven patients evaluated was 295 (± 87, range 187–409) minutes.

In Table 1, the mean value of various count rate variables, relative probe counting efficiency ratio, and T/B ratio for the nine 18F-FDG-avid tissue sites tested by the three different gamma detection probe systems are shown.

**Table 1:** Mean value of various count rate variables, relative probe counting efficiency ratio, and T/B ratio for the nine 18F-FDG-avid tissue sites tested by the three different gamma detection probe systems

**Mean value of each variable**	**K-alpha probe**	**RMD PET probe**	**Neoprobe PET probe**	**P-value**
**Averaged target tissue count rate (counts per second)**	960 (± 907, range 80–2509)	203 (± 153, range 45–446)	150 (± 121, range 32–322)	0.006
**Background tissue count rate in adjacent area of presumed normal tissue (counts per second)**	755 (± 858, range 32–2257)	133 (± 104, range 37–344)	71 (± 65, range 18–197)	0.014
**Relative probe counting efficiency ratio**	6.9 (± 4.4, range 2.2–15.4)	1.5 (± 0.3, range 1.0–2.1)	1.0 (± 0, range 1.0–1.0)	<0.001
**Calculated fixed T/B ratio**	1.6 (± 0.6, range 1.1–2.5)	1.6 (± 0.5, range 1.2–2.4)	2.3 (± 1.0, range 1.4–4.2)	0.073
**Calculated three-sigma criteria count rate (counts per second)**	827 (± 901, range 49–2400)	165 (± 117, range 55–400)	94 (± 76, range 31–239)	0.012

The mean of the averaged target tissue count rate for the nine ^18^F-FDG-avid tissue sites was 960 (± 907, range 80–2509) counts per second using the K-alpha probe system, 203 (± 153, range 45–446) counts per second using the RMD PET probe system, and 150 (± 121, range 32–322) counts per second using the Neoprobe PET probe system (P = 0.006).

The mean of the background tissue count rate in an area of presumed normal tissue within a region adjacent to the nine ^18^F-FDG-avid tissue sites was 755 (± 858, range 32–2257) counts per second using the K-alpha probe system, 133 (± 104, range 37–344) counts per second using the RMD PET probe system, and 71 (± 65, range 18–197) counts per second using the Neoprobe PET probe system (P = 0.014).

The probe counting efficiency was assessed for all three gamma detection probe systems. The mean relative probe counting efficiency ratio was 6.9 (± 4.4, range 2.2–15.4) for the K-alpha probe system, was 1.5 (± 0.3, range 1.0–2.1) for the RMD PET probe system, and was 1.0 (± 0, range 1.0–1.0) for the Neoprobe PET probe system (P < 0.001).

The mean of the calculated fixed T/B ratio for the nine ^18^F-FDG-avid tissue sites was 1.6 (± 0.6, range 1.1–2.5) for the K-alpha probe system, 1.6 (± 0.5, range 1.2–2.4) for the RMD PET probe system, and 2.3 (± 1.0, range 1.4–4.2) for the Neoprobe PET probe system (P = 0.073).

The mean of the calculated three-sigma criteria count rate for the nine ^18^F-FDG-avid tissue sites was 827 (± 901, range 49–2400) counts per second for the K-alpha probe system, 165 (± 117, range 55–400) counts per second for the RMD PET probe system, and 94 (± 76, range 31–239) counts per second for the Neoprobe PET probe system (P = 0.012).

The detection success rate for probe positivity for the nine separate ^18^F-FDG-avid tissue sites by the ratiometric threshold criteria method and by the three-sigma statistical threshold criteria method at the time of attempted *in situ* intraoperative detection was assessed for all three gamma detection probe systems. The K-alpha probe system detection success rate for probe positivity was in 3/9 cases (33%) by the ratiometric threshold criteria method and in 8/9 cases (89%) by the three-sigma statistical threshold criteria method. The RMD PET probe system detection success rate for probe positivity was in 3/9 cases (33%) by the ratiometric threshold criteria method and in 4/9 cases (44%) by the three-sigma statistical threshold criteria method. The Neoprobe PET probe system detection success rate for probe positivity was in 7/9 cases (78%) by the ratiometric threshold criteria method and in 8/9 cases (89%) by the three-sigma statistical threshold criteria method. Therefore, with each of the three gamma detection probe systems tested, successful *in situ* intraoperative detection of ^18^FDG-avid tissue sites was more frequently accomplished by using the three-sigma statistical threshold criteria method than by using the ratiometric threshold criteria method. While the overall categorical variable comparison of the three gamma detection probe systems utilized as a function of the specific threshold criteria used was not found to be statistically significant (P = 0.094), the individual categorical variable comparison of the K-alpha probe as a function of the specific threshold criteria used demonstrated that the three-sigma statistical threshold criteria method was significantly better than the ratiometric threshold criteria method for determining probe positivity for the K-alpha probe (P = 0.050). All other categorical variable comparisons for probe type as a function of threshold criteria and for threshold criteria as a function of probe type were found not to be statistically significant, with the lack of significant differences in these categorical variable comparisons most realistically attributable to the small number of cases available for each of these resultant 2 × 2 and 2 × 3 contingency table analyses.

The previously mentioned four intraoperative clinically suspicious sites that were identified in one of the seven patients (that were not ^18^F-FDG-avid on preoperative same-day diagnostic PET/CT imaging, but were intraoperatively assessed *in situ* with all three gamma detection probe systems and were subsequently surgical excised) were not determined to be probe positive by the ratiometric threshold criteria method or by the three-sigma statistical threshold criteria method at the time of attempted *in situ* intraoperative detection by any of the three gamma detection probe systems.

All nine separate ^18^F-FDG-avid tissue sites (which were identified on same-day preoperative diagnostic PET/CT imaging, and which were intraoperatively assessed *in situ* with all three gamma detection probe systems and subsequently surgical excised), were visualized as ^18^F-FDG-avid tissue sites on same-day perioperative *ex situ* specimen PET/CT imaging. The mean time from ^18^F-FDG injection to same-day perioperative specimen PET/CT imaging for the nine separate ^18^F-FDG-avid tissue specimens evaluated was 488 (± 130, range 340–661) minutes. None of the four intraoperative clinically suspicious sites that were identified in one of the seven patients (that were not ^18^F-FDG-avid on preoperative same-day diagnostic PET/CT imaging, but were intraoperatively assessed *in situ* with all three gamma detection probe systems and were subsequently surgical excised) were visualized as potential ^18^F-FDG-avid tissue sites on same-day perioperative *ex situ* specimen PET/CT imaging.

Final histopathologic evaluation of the nine separate ^18^F-FDG-avid tissue sites revealed squamous cell carcinoma of the head and neck region in five ^18^F-FDG-avid tissue sites, as well one site containing invasive ductal carcinoma of the breast, one site containing non-small cell carcinoma of the lung, one site containing malignant melanoma, and one site containing eccrine porocarcinoma. Final histopathologic evaluation of the four intraoperative clinically suspicious sites identified in one of the seven patients (that were not ^18^F-FDG-avid on preoperative same-day diagnostic PET/CT imaging and were not intraoperatively detected *in situ* with any of the three gamma detection probe systems and were subsequently surgical excised and were not visualized as potential ^18^F-FDG-avid tissue sites on same-day perioperative *ex situ* specimen PET/CT imaging) showed benign lymphoid tissue only.

## Discussion

It is our observation that *in situ* T/B ratios for ^18^F-FDG-avid tissue sites detected intraoperatively are often less than 1.5-to-1.0, making localization of such ^18^F-FDG-avid tissue sites very challenging when utilizing standard gamma detection probes and PET probes that rely solely on a fixed T/B ratio (ratiometric threshold criteria method) as the threshold for probe positivity. Therefore, an optimized gamma detection probe design that allows for the *in situ* intraoperative detection and localization of ^18^F-FDG-avid tissue sites having an *in situ* T/B ratio of less than 1.5-to-1.0 is essential to performing successful ^18^F-FDG-directed surgery.

In the current report, we evaluated the probe counting efficiency and the success rate of *in situ* intraoperative detection of ^18^F-FDG-avid tissue sites (using the three-sigma statistical threshold criteria method and the ratiometric threshold criteria method) for three gamma detection probe systems tested during ^18^F-FDG-directed surgery. We found that the mean relative probe counting efficiency was significantly better (P < 0.001) for the K-alpha probe system than for the two commercially-available PET-probe systems. Likewise, we found that successful *in situ* intraoperative detection of ^18^F-FDG-avid tissue sites was more frequently accomplished by using the three-sigma statistical threshold criteria method than by using the ratiometric threshold criteria method with each of the three gamma detection probe systems tested. In that regard, as based upon categorical variable comparison of the K-alpha probe as a function of the specific threshold criteria used, we specifically found that the three-sigma statistical threshold criteria method was significantly better than the ratiometric threshold criteria method for determining probe positivity for the K-alpha probe (P = 0.050). Yet, there was a general lack of significant differences in our analyses of all other individual categorical variable comparisons between probe type as a function of threshold criteria and between threshold criteria as a function of probe type. It is our contention this finding is most realistically attributable the small sample size (n = 9) that was available for the 2 × 2 and 2 × 3 contingency table analyses.

When applying commercially-available PET probe systems for the detection of ^18^F-FDG-avid tissue sites, the probe counting efficiency falls off rapidly with increasing gamma energy levels [19,20]. The intrinsic counting efficiency (i.e. the efficiency taking collimation and probe housing into account) of such commercially-available PET probe systems is less than 2% at a gamma energy level of 511 KeV [19,20]. The physical size and weight of a typical PET probe is primarily a function of the side shielding that is required to block background radiation, to limit the field of view, and to collimate the head of the probe, with the intention to limit the area of the tissue contributing to the probe count rate and to provide better spatial resolution between tissues of differing radioactivity levels [1]. Attempts at improving PET probe design by further increasing collimation and by creating crystal geometry of sufficient diameter and thickness to capture a higher percentage of 511 KeV gamma emissions would result in a PET probe construct that would be prohibitively large in physical size, heavy in weight, and expensive [1,21]. These factors represent significant barriers to the clinical application of currently commercially-available PET probe systems for the detection of ^18^F-FDG-avid tissue sites.

The use of collimation in PET probe design has very divergent effects on the probe counting efficiency versus the resultant T/B ratio observed, with collimation reducing probe counting efficiency at ^18^F-FDG-avid tissue sites and increasing the T/B ratio observed at ^18^F-FDG-avid tissue sites [1,19,20]. The effects of collimation are evident in both the determination of the probe counting efficiency and in the T/B ratio observed for the three gamma detection probe systems we examined, with the K-alpha probe having significantly better mean relative probe counting efficiency ratio as compared to the RMD PET probe system or the Neoprobe PET probe (6.9 for the K-alpha probe versus 1.5 for the RMD PET probe and 1.0 for the Neoprobe PET probe; P < 0.001) and with the Neoprobe PET probe having nearly-significantly improvement in the mean T/B ratio observed as compared to the K-alpha probe or the RMD PET probe (2.3 for the Neoprobe PET probe versus 1.6 for the K-alpha probe and 1.6 for the RMD PET probe system; P = 0.073). Therefore, the Neoprobe PET probe performed the best with the ratiometric threshold criteria method because it was specifically designed to maximize the T/B ratio through the use of increased collimation for attempting to directly count the 511 KeV gamma photon emissions. Yet, commercially-available PET probe systems, like the Neoprobe PET probe, which utilize increased collimation and have a resultantly low probe counting efficiency cannot fully take advantage of the three-sigma statistical threshold criteria method.

However, the K-alpha probe design [21], which lacks collimation, has a significantly higher probe counting efficiency, and has a decrease in the T/B ratio, can specifically benefit from the use of the three-sigma statistical threshold criteria method. It is our contention that the higher probe counting efficiency of the K-alpha probe design allowed for successful *in situ* intraoperative detection of ^18^F-FDG-avid tissue sites with lower T/B ratios, even down to a T/B ratio as low as 1.1-to-1.0. This is the end result of the fact that the K-alpha probe [21] does not directly count the 511 KeV gamma photon emissions, and instead counts the secondary, lower energy gamma emissions (K-alpha x-ray fluorescence) from a thin lead plate placed between the detection crystal and the source of gamma emissions, producing a much higher probe counting efficiency. Thus, its higher probe counting efficiency and the direct counting of secondary, lower energy gamma emissions by the K-alpha probe lends well to maximizing the benefits from use of the three-sigma statistical threshold criteria method. Furthermore, the K-alpha probe can be designed to be significantly smaller and lighter in weight than any commercially-available PET probe system, since the detection crystal can be made relatively thin and can be housed within a detection probe head with little or no needed collimation [21]. This resultant K-alpha design opens up the possibilities for the production of a commercially-available PET probe system that can be easily adapted for use in laparoscopic and robotic surgeries.

## Conclusions

Probe counting efficiency was significantly better for the K-alpha probe system than for the two commercially-available PET-probe systems. Successful *in situ* intraoperative detection of ^18^F-FDG-avid tissue sites was more frequently accomplished with each of the three gamma detection probe systems tested by using the three-sigma statistical threshold criteria method than by using the ratiometric threshold criteria method, specifically with the three-sigma statistical threshold criteria method being significantly better than the ratiometric threshold criteria method for determining probe positivity for the K-alpha probe. Our results suggest that the improved probe counting efficiency of the K-alpha probe design used in conjunction with the three-sigma statistical threshold criteria method can allow for improved detection of ^18^F-FDG-avid tissue sites when a low *in situ* T/B ratio is encountered. Further research and development are needed to more clearly understand these findings and to optimize gamma detection probe design for the intraoperative detection of ^18^F-FDG-avid tissue sites during ^18^F-FDG-directed surgery.

## Competing interests

Gregg J. Chapman has equity in Navidea Biopharmaceuticals and is a paid consultant for Dynasil Corporation; however, he reports no conflicts of interest with regards to the conduct of this study.

Edward W. Martin, Jr. has equity in Actis, Ltd and Navidea Biopharmaceuticals; however, he reports no conflicts of interest with regards to the conduct of this study.

All the other authors declare that they have no competing interests to report.

## Authors’ contributions

**SPP** was responsible for the overall study design, data collection, data analysis and interpretation, writing of all drafts of the manuscript, and has approved the final version of the submitted manuscript. **GJC** was involved in study design, data interpretation, writing portions of the manuscript, and has approved the final version of the submitted manuscript. **DAM** was involved in study design, data collection, data analysis and interpretation, writing portions of the manuscript, and has approved the final version of the submitted manuscript. **RL** was involved in study design, data interpretation, writing portions of the manuscript, and has approved the final version of the submitted manuscript. **EWM** was involved in study design, critiquing drafts of the manuscript, and has approved the final version of the submitted manuscript. **NCH** was involved in study design, data interpretation, writing portions of the manuscript, and has approved the final version of the submitted manuscript.

## Pre-publication history

The pre-publication history for this paper can be accessed here:

http://www.biomedcentral.com/1471-2407/13/98/prepub
